# Fatal congenital Chagas' disease in a non-endemic area: a case report

**DOI:** 10.1186/1757-1626-1-302

**Published:** 2008-11-07

**Authors:** María Flores-Chávez, Yamile Faez, José M Olalla, Israel Cruz, Teresa Gárate, Mercedes Rodríguez, Pilar Blanc, Carmen Cañavate

**Affiliations:** 1 Servicio de Parasitología, Centro Nacional de Microbiología, Instituto de Salud Carlos III, Ctra. Pozuelo-Majadahonda Km 2, 28220 Majadahonda, Madrid, Spain; 2 Hospital Carlos Haya, Av. Carlos Haya s/n, 29010 Málaga, Spain

## Abstract

The early diagnosis of congenital Chagas' disease is very important if infected newborns, whether symptomatic or not, are to receive adequate treatment. This paper describes the complications arising in the diagnosis of a newborn with fatal congenital Chagas' disease in Spain, a non-endemic area where visceral leishmaniasis is present.

## Introduction

Chagas' disease is endemic from the south of the United States to southern Argentina and Chile, where it is mainly transmitted by reduvid insects. The protozoan that causes the disease – which has a wide range of clinical manifestations – is *T. cruzi*, a parasite that shows great genetic variation. Large numbers of protozoa are detected during the acute phase of the disease, while numbers are much lower during the chronic phase. However, it is during this latter phase when the humoral immune response is patent.

Vertical or congenital transmission of *T. cruzi *is also possible. This route is associated with an infection rate of 0–10%, depending on the geographical area in question. Recent studies indicate the majority of children born to infected mothers to be asymptomatic, although some 2–10% present with severe respiratory distress, hepatosplenomegaly, myocarditis and meningoencephalitis [1]. Without specific treatment, the mortality rate among such children is high. Since the transmission of *T. cruzi *during pregnancy cannot be prevented, early diagnosis in newborns is essential so that appropriate etiological treatment can be administered; such treatment can be 100% effective. Since maternal IgG antibodies and excretion-secretion antigens that stimulate IgM or IgA production can cross the placenta [2], serological tests cannot discriminate between infected and non-infected newborns. However, the presence of anti-*T. cruzi *antibodies in maternal serum identifies mothers whose newborns are at risk of having been infected [2]. Accordingly, to confirm the parasite infection, mobile *T. cruzi *trypomastigotes should be sought microscopically in the cord blood or peripheral blood of newborns after a concentration procedure (the microhaematocrit test) [3].

## Case presentation

**Figure 1:**
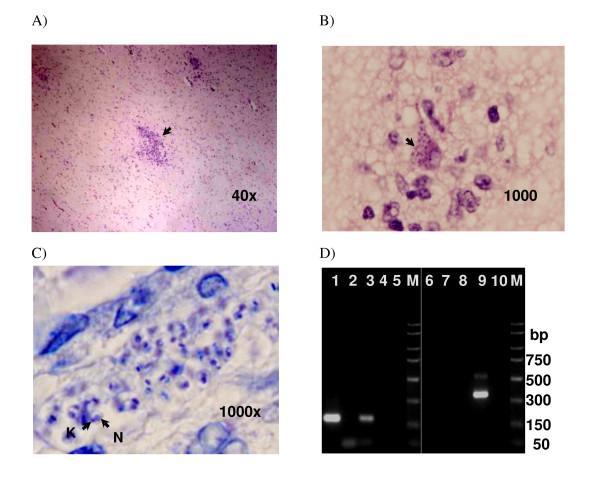
Detection of *Trypanosoma cruzi *parasites in samples of the deceased child. A and B, sections of cerebral cortex stained with hematoxylin and eosin, and C, brain stem stained with Giemsa stain. The magnification is shown in the lower right corner; C is digitally magnified. Arrows indicate: in A, an inflammatory nodule, in B, parasites in the cytoplasm, and in C the nucleus (N) and kinetoplast (K) of one of the amastigotes. D, Results of Tcz1-Tcz2 PCR (lanes 1 to 5) and Ln-PCR (lanes 6 to 10) from serum. Lanes 1 and 6, serum; lanes 2, 5, 7 and 10, negative controls; lanes 3 and 8, *T. cruzi *positive control; lanes 4 and 9, *Leishmania *positive control; M, molecular weight markers (PCR marker 50 – 2000 bp).

A 37 year-old Argentine woman with a background of bipolar disease attended an appointment for a fetal ultrasound scan during the third trimester of pregnancy at a teaching hospital. The results revealed her unborn child to be suffering a 3–4 weeks delay in growth, unilateral ventriculomegaly, and signs of stress. Birth was induced at 34 weeks (delivery was by Caesarean section); the newborn weighed 1130 g and was in a convulsive state. Physical examination of the child revealed scant spontaneous activity, moderate responses to stimuli, poor peripheral perfusion, severe microcephaly, small palpebral slits, foci of choroiditis, vitreous opacity, and hepatosplenomegaly. Cranial ultrasonography revealed a left subependimal hemorrhage and dilation of the occipital horns of the lateral ventricles. A cranial computed tomography (CT) scan confirmed the dilation of the lateral ventricles and revealed colpocephaly and megacisterna magna. Electroencephalography indicated a low to moderate activity voltage and discrete signs of dysrhythmia in the frontal zone. Ultrasonography detected hypertrophy of the left ventricle of the heart; systolic function was conserved. In abdominal ultrasound scans the liver was hyperechogenic; moderate hepatosplenomegaly was also seen. Blood tests revealed leukopenia, neutropenia and persistent thrombocytopenia, hypoproteinemia, hyponatremia and hypocalcemia, elevated non-conjugated bilirubin levels, elevated concentrations of glutamyl oxaloacetic transaminase, glutamyl pyruvic transaminase, gamma-glutamyl transpeptidase and alkaline phosphatase. To rule out a possible intrauterine infection or connatal sepsis, blood and cerebrospinal fluid (CSF) were cultured with negative results. The latex agglutination test for *Streptococcus *group B bacteria and the shell-vial test for cytomegalovirus were performed on urine samples; all results were negative. Serological tests ruled out TORCHES, Epstein-Barr virus, parvovirus (B19), enterovirus and adenovirus infections. During the newborn's stay in the intensive care unit, fever peaks were recorded. A lower urinary tract infection caused by *Escherichia coli *was identified; treatment with ampicillin and cefotaxime was administered. Newborn blood, urine, fecal and CSF samples were examined by the *Centro Nacional de Microbiología *(CNM), a Reference Center for Infectious Diseases for the Spanish Healthcare System, to test for viral infections and toxoplasmosis; all tests were negative. Tests of samples of maternal serum ruled out rubella, cytomegalovirus, syphilis, toxoplasmosis and HIV; only anti-herpes IgG antibodies were detected (IgM negative). The child died after 2 1/2 months of poor clinical progress during which no response to symptomatic treatment was seen. Autopsy revealed generalized nodular encephalitis. The entire cerebral cortex showed inflammatory nodules around areas of necrotic tissue (Fig. 1A). The cytoplasm of the mononuclear phagocytes in the cortex and brainstem showed round structures compatible with Leishman-Donovan bodies (Fig. 1B and 1C). The same structures were not seen, however, in histological sections of the myocardium, liver or spleen. Taking into account these results, the *post mortem *diagnosis was leishmaniasis. To investigate the possible route of *Leishmania *infection, the mother and the rest of her family underwent serological tests for this pathogen. A commercial indirect immunofluorescent antibody test (IFAT) kit (Leishmania Spot IF, bioMérieux) detected IgG anti-*Leishmania *antibodies in the mother and one of the child's two siblings (Table 1).

**Table 1:** Detection of anti-*Trypanosoma cruzi *and anti-*Leishmania *antibodies in the family members of the deceased child.

	*T. cruzi *tests		*Leishmania *tests		
		
	In-house^a ^ELISA	In-house^b ^IFAT	rk39-ELISA^a^	In-house^b ^IFAT	Commercial^b ^IFAT
Mother	2.09	> 1/320	0.04	N	1/1280
Father	0.05	N	0.01	N	N
Brother 1	2.10	1/320	0.07	± 1/80	1/320
Brother 2	0.04	N	0.01	N	N
Deceased child	1.89	> 1/320	0.03	N	nd

NOTE. - ^a^Results are expressed as absorbances. The cut-off in the *T. cruzi *ELISA test was 0.5, and 0.2 in the rk39 ELISA test.

^b^The IFAT results show the serum dilutions in which fluorescent parasites were observed. The cut-off for *T. cruzi *was 1/40, and 1/80 for *Leishmania*. N = negative, ± = parasites observed with and without fluorescence, nd = not done.

To confirm these latter results, sections of the child's brain, spleen, liver and heart embedded in paraffin wax were examined by the Parasitology Department of the CNM. The serums of the family members (stored at -20°C until use) were also analyzed. DNA was extracted from the embedded tissues and from the child's serum, and PCR was performed using oligonucleotides R221-R332 and R223-R333 (Ln-PCR) [4] to test for *Leishmania *spp., and Tcz1-Tcz2 [5] to test for *Trypanosoma cruzi*. The latter pathogen was detected in all newborn's samples examined, but *Leishmania *was not detected. The *T. cruzi *genotype was determined by PCR of miniexon and 24Sα rRNA genes [6,7], Tc IId was identified. In addition, in-house IFAT using *L. infantum *as antigen showed an absence of anti-*Leishmania *antibodies in all family members tested, except for a doubtful positive result for one of the deceased child's siblings. Further testing found no antibodies to rk39 [8], a specific antigen of the *Leishmania donovani *complex, in any family member. IgG anti-*T. cruzi *antibodies were detected by IFAT and ELISA (using *T. cruzi *as antigen) in the mother and in the same sibling as above – a clear marker of *T. cruzi *infection. Anti-*T. cruzi *antibodies were also found in serum of the newborn (Table 1).

## Discusion

In Spain, Chagas' disease is an emerging imported parasitosis. In recent years, the population of immigrants from areas where the disease is endemic has grown notably; indeed, these citizens represent some 34% of the country's entire immigrant population (1 735 025 are Latin Americans) [9]. This situation has influenced legislation regarding the selection of blood donors; thus since 2005 Spanish law requires all blood transfusion centers to perform validated testing to rule out *T. cruzi *infection on individuals with epidemiological risk. However, no consensus has been reached with respect to the monitoring of pregnant women from endemic areas, and only some Spanish regions (Valencia, Catalonia and Murcia) perform serological screening during pregnancy. To date, a number of cases of congenital transmission have been described, in which the administration of appropriate treatment was associated with favorable clinical progress [10,11].

The present paper describes a severe case of congenital Chagas' disease that was diagnosed *post mortem*. This highlights the limitations of conventional microscopic and serological analyses when the epidemiological background is not considered. Since information on the mother's origin was not made available while the child was alive, the possibility of Chagas' disease was not taken into account, therefore the newborn was monitored following the normal Spanish protocols.

The *post mortem *detection of *T. cruzi *DNA in a serum sample from the newborn analyzed at the Parasitology Department of the CNM indicates that parasites had been circulating in the peripheral blood (Fig. 1D); a lack of experience in microscopical diagnosis of trypomastigotes probably explains why they were unnoticed in earlier blood tests. On the other hand, as the tissue amastigotes of *T. cruzi *are morphologically very similar to the *Leishmania *amastigotes, they can be confused in microscopic examinations. With respect to immunodiagnosis, although the conventional serology procedures for both protozoa show good sensitivity and specificity in terms of discriminating between infected and non-infected individuals, cross reactivity is a problem. The present data show that the commercial IFAT kit used to test for anti-*Leishmania *antibodies suffers this disadvantage (Table 1). Therefore, the reliability of commercial serological kits for the diagnosis of Chagas' disease and leishmaniasis should be assessed in this new epidemiological context, especially since parasitological diagnosis is of low sensitivity in the chronic phase of *T. cruzi *infection. It should be remembered that the recombinant antigen rk39 shows no cross reactivity with sera from patients with Chagas' disease [8], therefore the absence of anti-rk39 antibodies in any of the newborn's family members confirms that what were thought to be anti-*Leishmania *antibodies detected by the commercial IFAT kit were a product of cross-reactivity. Since in Spain, the immigrant population is exposed to *Leishmania *infection, and given that cross reactivity can be a problem in serological analyses, PCR might offer the best way to discriminate between *L. infantum *and *T. cruzi*. Indeed, from year 2002 to 2007, 100 newborns' blood samples (whose mothers were seropositive to *T. cruzi *infection) were examined by *T. cruzi*-PCR at the Parasitology Department of CNM-ISCIII. It is important to highlight that three new cases were PCR positive in the last year (3/78).

Neither the detection of parasites in the placenta nor maternal infection with any particular genotype are indicators of congenital infection. However, it has been reported that mothers who transmit the *T. cruzi *infection commonly have high parasitaemia and show an associated reduction in γ-IFN production [12]. Although these factors were not examined in the present mother, it is probable that a state of immunodepression had been favored by her bipolar disease, which in turn would have favored a high parasite load and the infection of her fetus. The *post mortem *microscopical detection of the parasite in the child's brain tissue might explain the ultrasonography and cranial CT findings. Although no amastigotes were seen in the remaining tissues examined, the presence of *T. cruzi *DNA in all the necropsy samples suggests that the parasite was distributed all over the child's body, causing the pathological alterations recorded. At monitoring times during the first and second trimester, ultrasound scans revealed no fetal abnormalities, suggesting that intrauterine infection occurred during the third trimester.

## Conclusion

In the absence of prognostic markers for the development of the infection, a procedure involving i) the serological screening of pregnant women from Latin America, ii) fetal monitoring by ultrasonography, and iii) parasitological diagnosis of the newborn, via direct microscopic observation and molecular tests, may contribute towards the early detection of congenital *T. cruzi *infection in non-endemic areas, allowing newborns to receive timely treatment. Moreover, since reinfection is unlikely in Spain, providing post-partum anti-parasite treatment to infected mothers could help avoid further instances of vertical transmission.

## Consent

Written informed consent was obtained retrospectively from the patient's mother for publication of this case report and accompanying images. A copy of the written consent is available for review by the Editor-in-Chief of this journal.

## Competing interests

The authors declare that they have no competing interests.
